# An innovative traffic light recognition method using vehicular ad-hoc networks

**DOI:** 10.1038/s41598-023-31107-8

**Published:** 2023-03-10

**Authors:** Esraa Al-Ezaly, Hazem M. El-Bakry, Ahmed Abo-Elfetoh, Sara Elhishi

**Affiliations:** 1 Information Systems Department, Faculty of Computer and Information Sciences, Mansoura University, Mansoura, Egypt; 2 Head of Information Systems Department, Faculty of Computer and Information Sciences, Mansoura University, Mansoura, Egypt

**Keywords:** Mathematics and computing, Electrical and electronic engineering, Network topology

## Abstract

Car congestion is a pressing issue for everyone on the planet. Car congestion can be caused by accidents, traffic lights, rapid accelerations, deceleration, and hesitation of drivers, as well as a small low-carrying capacity road without bridges. Increasing road width and constructing roundabouts and bridges are solutions to car congestion, but the cost is significant. TLR (traffic light recognition) reduces accidents and traffic congestion caused by traffic lights (TLs). Image processing with convolutional neural network (CNN) lakes dealing with harsh weather. A semi-automatic annotation for traffic light detection employs a global navigation satellite system, raising the cost of automobiles. Data was not collected in harsh conditions, and tracking was not supported. Integrated channel feature tracking (ICFT) combines detection and tracking, but it does not support sharing information with neighbors. This study used vehicular ad-hoc networks (VANETs) for VANET traffic light recognition (VTLR). Information exchange as well as monitoring of the TL status, time remaining before a change, and recommended speeds are supported. Based on testing, it has been determined that VTLR performs better than semi-automatic annotation, image processing with CNN, and ICFT in terms of delay, success ratio, and the number of detections per second.

## Introduction

Traffic jams are increasing at high rates in all countries, particularly at intersections. At intersections, traffic lights force drivers to accelerate suddenly when the traffic light turns green. This leads to high fuel consumption and air pollution. Drivers’ hesitation to stop or go to yellow traffic lights can cause accidents^1^.

Early information on the traffic light status of a driver reduces the number of stops and urgent acceleration. It also reduces accidents, traffic jams, and pollution, which increase at intersections^2^. Intelligent traffic organization at an intersection is required. Smart traffic lights can be used to support traffic information and smart driver decisions^3^. Communication between the driver and smart traffic lights is required.

It is important to detect traffic lights in order to reduce the number of accidents caused by TL systems. In addition, TLRs reduce the number of accidents and crowded vehicles. Traditional detection methods using GPS, cameras, and sensors have many problems and trade-offs. Many TLs remain undetected owing to the time difference between the detector, tracker, classifier, camera, and real-time. Therefore, many of these devices must communicate with each other and the host to avoid this time lag. VANET supports several vehicles with massive data rates^4^. Lamps and other light sources can yield false-positive results. TL occlusion occurs because of harsh weather conditions or excessive lighting^5^.

Previously, the traffic light controller was a one-purpose device with proprietary hardware and software. There were no methods for adding or communicating with other applications. Modern traffic light controllers are based on Linux and use faster processors. It allows access to shared controller resources and supports many applications^6^. The digital TL contains a counting-down clock until a change is detected, to prevent driver hesitation.

Wireless networks have recently been used to direct roads and traffic. A vehicular ad hoc network (VANET) is a wireless network that allows easy communication between vehicles^7^. Based on VANET, smart traffic lights can disseminate important traffic light information, such as current and next traffic light status, and appropriately advised speeds for vehicles. Vehicles can then disseminate information to their neighbors. Smart traffic lights help to reduce traffic jams and pollution by reducing stops and urgent acceleration at intersections. It also reduces accidents caused by urgent stops, acceleration, and deceleration^4^.

Communication between the TL controller and vehicle was established using DSRC radio^8^. The communication types in VANETs are divided into three categories that are strongly related to VANET components:

1. Vehicle to vehicle (V2V) communications A technology that allows vehicles to communicate with each other through multi-hop messages is exchanged to allow different services and applications.
2. Vehicle to infrastructure (V2I) or vehicle roadside (V2R) communications The vehicle communicates with roadside infrastructure installed along the road to provide services and user information.
3. Roadside-unit to roadside-unit (R2R) communications R2R technology allows RSUs to communicate with each other to provide various services to vehicles. For example, when two vehicles move farther away from each other and cannot communicate directly, RSUs are used. R2R communication plays a vital role in both V2V and V2R communications in verifying the identity of the vehicles that pass them. RSUs can also exchange location and traffic information to control driving. Thus, it guarantees a better organization.

This study proposes a novel approach to traffic light recognition. Traffic lights communicate their status via VANET to nearby neighbors like RSUs and vehicles. The color of the current and upcoming traffic light signal is communicated to neighbors. Traffic light (TL) controllers and vehicles were both linked to VANET. The suggested current speed can also be provided to drivers.

In this study, the LISA dataset was employed because it contains certain features. The LISA dataset contains thousands of traffic light images from continuous tests. The LISA dataset is widely used for traffic light detection. The sequences were captured using a stereo camera mounted on the roof of the vehicle while traveling at night and during the day. The dataset shows various lighting and weather conditions.

The remainder of this paper is organized as follows. Section “Related work” discusses related work reported in the literature. Section “Traffic light recognition” provides a detailed explanation of the proposed VTLR. In Sect. “Performance metrics”, the recommended speed of VTLR is evaluated analytically. In Sect. “Results and evaluation”, the proposed model is tested based on analytical and SUMO/OMNET +  + simulation results. Finally, Sect. “Conclusion” concludes the paper.

## Related work

Traffic light detection and recognition require several support techniques. Lidar, radar, and GPS are expensive for rebuilding traffic light control systems and increasing privacy issues and vehicle prices. Cameras face many difficulties such as high traffic lights and sunlight. The sensor and camera suffer from blinding spots because of cars, buildings, and signs^9^.

The target traffic light is small and ambiguous because of weather and light^10^. Therefore, two ways were used; The first technique is a vision-based traffic-light structure detection and convolutional neural network (CNN) based state recognition system that is robust in various lighting and weather circumstances^11^. Neural networks have been trained using many green and red signal samples for traffic-light state detection. The data contained videos with more complex scenes, and the imaging quality of the traffic lights was unstable. The threshold was used to extract the HSV color, and the kernel was used to describe the candidate regions of the traffic light. An adaptive background suppression filter was implemented to predict the location of traffic lights. The second method employs a deep fusion network to accomplish robust fusion without requiring a large corpus of labelled training data that covers all asymmetric distortions^12^.

Traffic light also has a timer of seconds down to the red and green traffic. Traffic-light character recognition is achieved through important steps of segmentation, feature extraction, and classification. Recent studies have examined the lake timer detection, recommended travel speed dissemination, and traffic-light status information dissemination^13^.

Previous studies on traffic-light recognition have been based on neural networks. A semi-automatic annotation for building traffic datasets was proposed. A training neural network for traffic light detection was used; however, it lacked tracking. Global Navigation Satellite System (GNSS) and inertial navigation system (INS) are integrated, which increases the cost of cars^14^. Numerous samples of traffic lights were randomly identified as positive samples, and many samples as negative samples were identified among the traffic light samples (non-traffic light samples). Non-traffic lights have a more varied structure than traffic lights do. As a result, the number of non-traffic light samples used as TL detection training data is substantially greater. Data under harsh conditions such as cloudy and rainy weather were not collected.

Integrated channel feature tracking (ICFT) combines detection and tracking using a convolutional neural network. A total of 80,000 traffic-light images were obtained under different weather conditions. A large dataset increases storage^15^. A 12 GB memory computer was used. However, information-sharing with neighboring vehicles is not supported.

A heuristic candidate region selection module was used to identify all possible traffic lights, and a lightweight Convolution Neural Network (CNN) classifier was used to classify the results^16^. Multi-sensor data (GPS, camera, and LiDAR) were collected in a normal environment^17^. The adaptively dynamic adjustment (ADA) model was developed by analyzing the relationship between the sensor error and the optimal ROI size. Furthermore, the optimal ROI for predicting the traffic light location can be obtained using the multi-sensor data fusion positioning and the ADA model. Finally, the image features were extracted and identified using YOLOv4. Daytime was not considered. The proposed algorithm is tested using a public dataset and nighttime city road test.

The LISA traffic light dataset was used to conduct separate detection and recognition experiments^18^. An improved YOLOv4 algorithm was used. Image processing with C# programming and color detection methods have been used^19^. The captured image was processed in two stages: preprocessing to detect markings and Gaussian filtering. However, recognition must be performed in real-time to ensure safety.

The Hough Circle Transform image processing technique was used to detect the red and green circles of traffic lights^20^. The efficiency of the technique in terms of improved time and accuracy was demonstrated on a real dataset, which includes various illumination conditions, such as day, evening, night, cloudy weather, and rain. Large trunks and signs in building blocks were not considered.

There are some problems and difficulties associated with previous studies. Recognition must operate in complex and changing weather and traffic environments. This must be performed in real-time to ensure the safety of the vehicle while driving. Tracking traffic light information must be combined with detection. Harsh weather conditions, such as mist, fog, rain, dust, smoke, and storms must be considered. Images taken at midday contain the reflection of the windshield owing to intense sunlight. Large trunks and signs in the building blocks when traffic lights are invisible must also be considered. Different two-way road illuminations must be considered. Timer detection is required for TL prediction. The TL time and status must be disseminated to other vehicles to enhance traffic.

## Traffic light recognition

In this study, an innovative traffic light recognition method is proposed. VANET is used to send traffic light status from Traffic light to neighbors such as RSUs and vehicles. Current and next traffic light signal colors are disseminated to neighbors. Vehicles and traffic light (TL) controllers were connected to VANET. Drivers can be informed of their current TL status, time to change, next TL status, and a recommended current speed. The dissemination of this information is also allowed to use VANETs.

**Figure 1:**
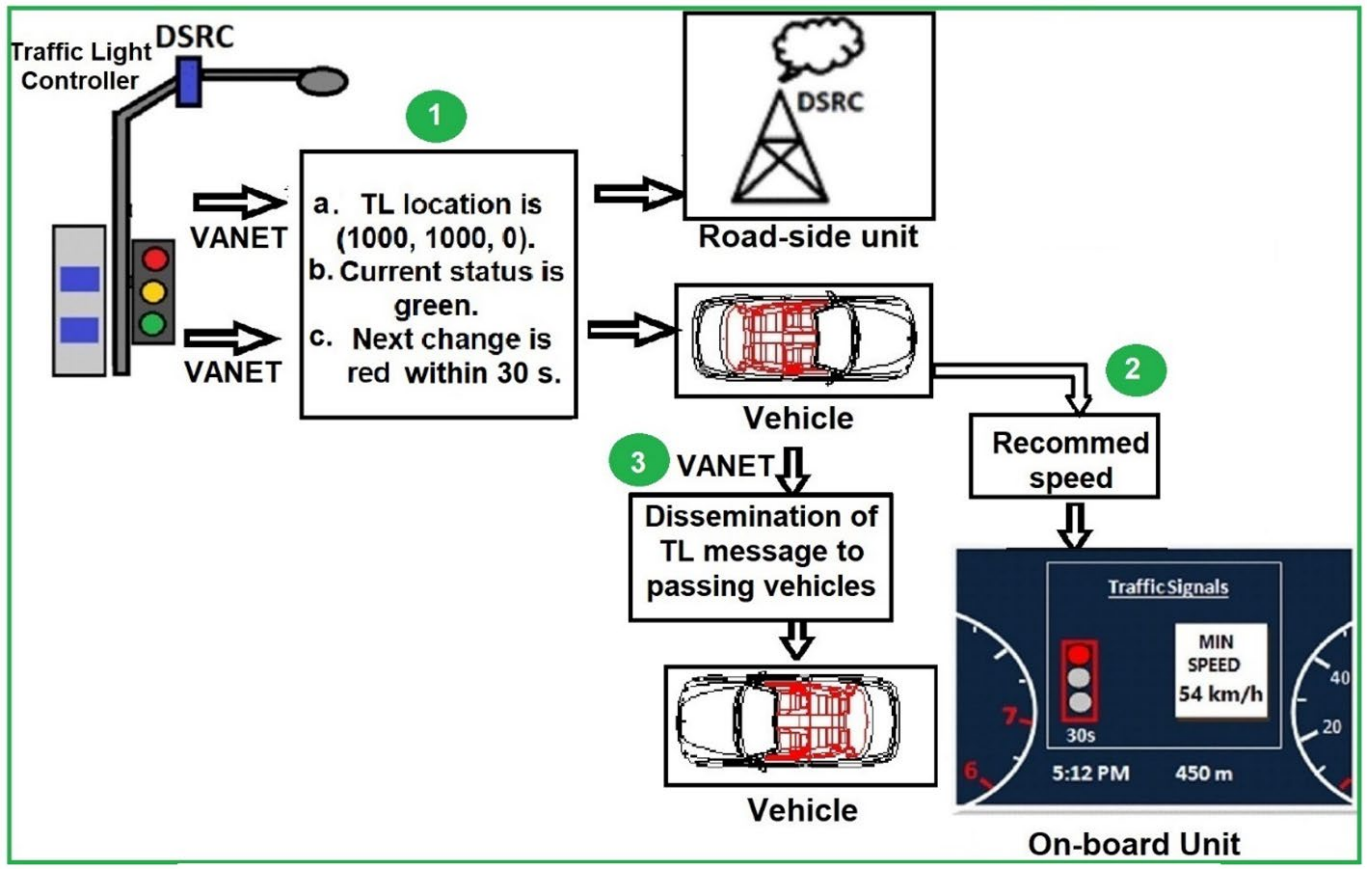
A framework for traffic light recognition using VANET.

A framework for traffic light recognition using VANET is shown in Fig. 1. This framework proposes an efficient system that includes:

1. TL recognition using dissemination
  - Location of current TL bounding box.
  - Current TL status.
  - Clock for time to change.
2. Recommended vehicle speed.
3. Dissemination of neighbors.

**Figure 2:**
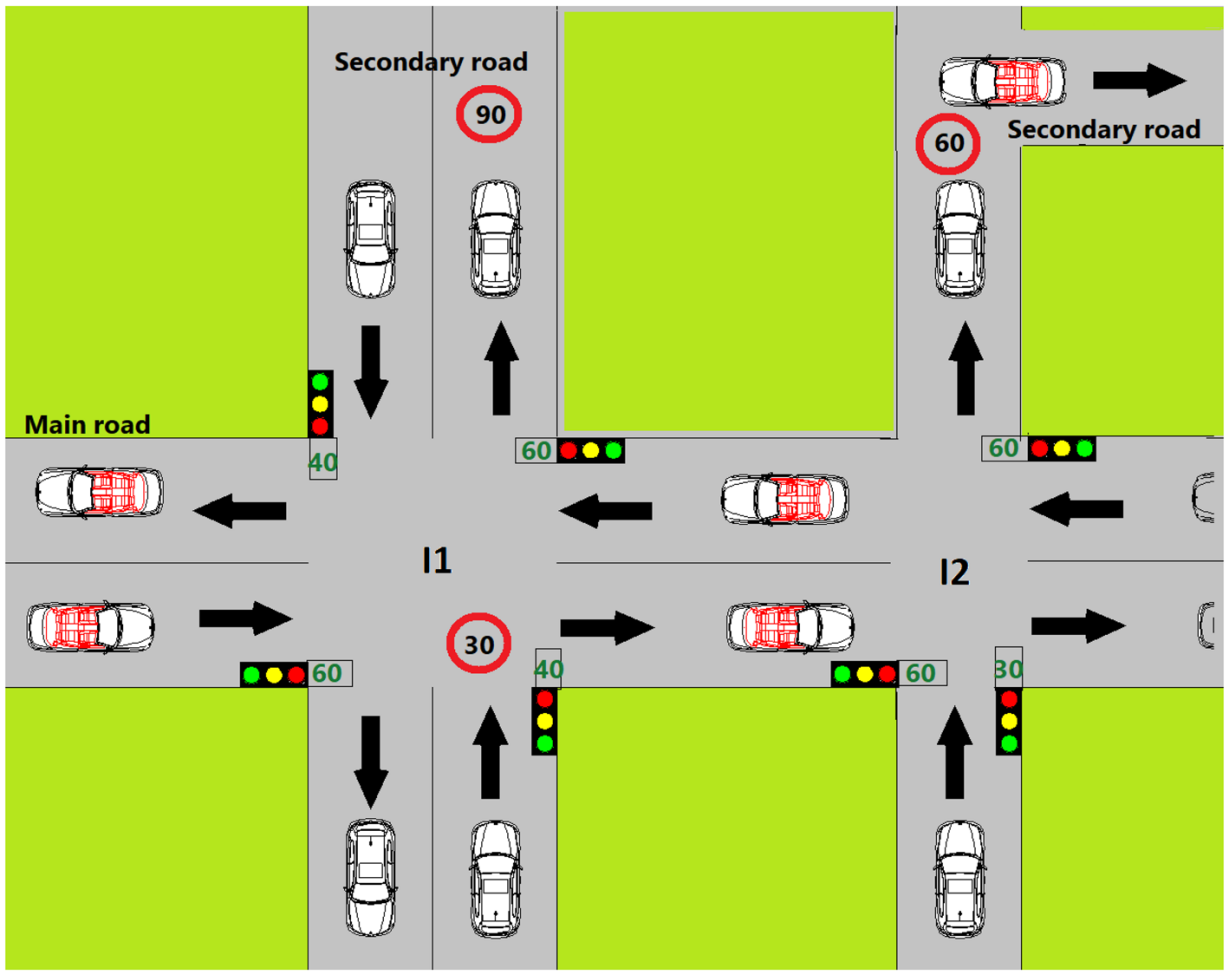
VANET-enabled traffic light boxes in each road direction.

Traffic lights are used to disseminate TL position, time, and status using a VANET. The TL works as RSU. In Fig. 2, the TL and vehicles communicate using DSRC and are divided into three sections. In Sect. “Traffic light recognition”, the traffic light recognition and waning dissemination algorithm using a VANET are described. In Sect. “Recommended vehicle’s speed”, the prediction of the recommended vehicle speed is presented. In Sect. “Dissemination”, message dissemination to neighbors is discussed.

### Traffic light recognition

There are two types of roads: main and subway roads. In Fig. 2, it is assumed that the road intersection is large and that the main road has a faster average speed (90 km/h) than the subway (60 km/h). In popular TL, the light status is green, yellow, red, or green. Each road type had two opposite directions. Therefore, there are four directions. Each direction had a traffic light box on the side of the intersection. If it is assumed that the main road lasted 40 s until the green light changed to red, it is assumed that this time was more than 30 s until the green light changed to red on the subway road.

### Recommended vehicle’s speed

**Figure 3:**
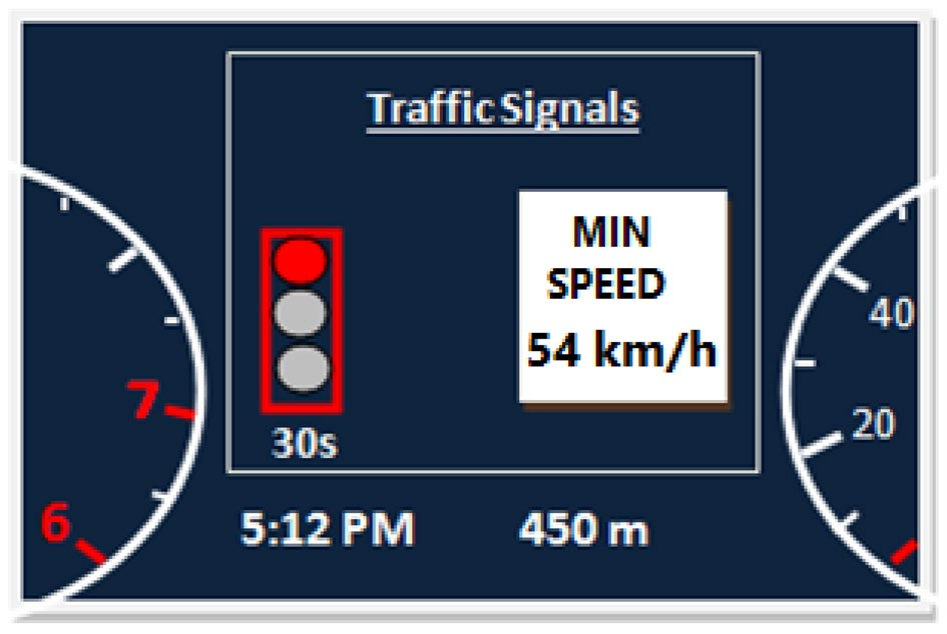
The traffic light signal information appears on the dashboard of the vehicle.

The traffic light signal information displayed in the vehicle is the current signal state and the time until change. The indicator in the instrument panel shows the countdown timer with the predicted time taken to go green. It is blank if it cannot be predicted. The data analysis predicted the best-recommended speed. In Fig. 3, traffic light information appears on the dashboard of the vehicle. Traffic light color, the timer of seconds until changes, and currently recommended speed appear on the dashboard.

The predicted duration, which is the suggested speed message duration, suggested speed, and suggested speed message time, was calculated using Eqs. (1), (2), and (3), respectively. At each prediction duration, the suggested message is sent to the driver on the dashboard. Equation (1) shows when to suggest an appropriate speed for the driver. The suggestion time was measured in seconds. The forecast time depends on the number of messages sent during the time between traffic signal changes.

1 $${D}_{P}=\frac{({T}_{E}-{T}_{B})}{N}.$$

${D}_{P}$ is the prediction duration (s), ${T}_{E}$ is the TL change time, ${T}_{B}$ is the prediction start time, and N is the number of messages. The suggested speed was the distance from the current position of the car to the traffic light. Velocity equals distance over time. The predicted distance and time are small, and close to intersections. The distance is not linear in a free space such as mobile ad-hoc networks.

While vehicles in VANET have constrained mobility and can only travel along the paved road network, nodes in mobile ad hoc networks are assumed to move freely in any direction. The topology of the network changes quickly in the VANET because nodes are moving vehicles^7^. Computing the distance between the vehicle and the traffic light is made easier with the aid of a digital road map. The road digital map is mapping earth coordinate into map pixels to calculate road distance. The predicted distance is computed for the real paved road from the vehicle to the traffic light using road map in the simulator used in testing to guarantee non-linear nature of VANET model^22^. Vehicular network simulation (Veins) is built on top of OMNET +  + and simulates urban mobility (SUMO). In SUMO, distance is calculated using Time, in the proposed algorithm, is not affected by traffic jam delays because all drivers are advised of the recommended speed which guarantees low density and low jams at traffic lights and intersections. Therefore, there is no need for adding prediction delay to computations.

The driver should pass quickly before the traffic light turned red. However, this must not exceed the speed of the road. Otherwise, a warning must be sent to the driver. If the suggested speed exceeds the road speed, a slowdown warning is sent until the driver stands at a red traffic light. To convert from meters per second to kilometers per hour, the speed is multiplied (1000/(60 × 60)) as in Equation 2.

2 $$\mathrm{Sc}=\frac{D*3600}{{T}_{R}*1000}.$$

S_c_ is the suggested speed (km/h), D is the distance to the traffic light and ${\mathrm{T}}_{\mathrm{R}}$ is the time that remains until TL changes. Where S_c_ must not exceed the road speed. If the speed exceeded the road speed, a slow-down warning was sent to the driver. If the suggested speed was higher than the road speed, then the next TL status time was added to the remaining time as in Equation 3.

3 $${T}_{R} ={T}_{R}+ {T}_{S}.$$

$${T}_{R} is$$ the time remaining until the traffic light changes (s) and $${T}_{S}$$ is the next TL status time. Algorithm 1 shows the message sent to the dashboard of the vehicle to suggest speed limits to the driver. Traffic light enabled VANET to send time until change ($${T}_{R})$$ and distance to TL (D) to its neighbors in step 2. If the TL status is red, the minimum speed is provided in Step 7. If the suggested speed was higher than the road speed, the next TL status time (T + 1) was added to the remaining time ($${T}_{R}$$). If the TL status is green, then the minimum speed is provided in step 14. If the vehicle is far from the TL and the suggested speed is low, another road speed is calculated in step 23. Otherwise, the suggested speed message is sent to the vehicle using VANET in Step 25 to advise the driver. Table 1 shows an example row from a group member table extracted from beacon messages sent from vehicles N1, N2, and N3 to the TL. The table contains the information recording time (s) for the recorded vehicle number, as well as the location of the source vehicle of the beacon message at the x, y, and z coordinates, speed, road number, and road type. Table 2 lists the notation of the algorithm.

**Table 1:** Example of a group member table.

Time (s)	Vehicle number	Location (x, y, z)	Speed (m/s)	Road type
15	N1	1500, 1500, 0	10	Primary
16	N2	1500, 1600, 0	15	Primary
17	N3	1600, 1600, 0	10	Secondary

**Table 2:** Notations of algorithm 1 and their descriptions.

Notation	Description
K	Number of vehicles
I	Number of available road branches
Min S_s_	The suggested minimum speed
Max S_s_	The suggested maximum speed
D	Distant to TL
T	Status time
S_max_	Max road speed
${\mathrm{T}}_{\mathrm{R}}$	Time until TL status changes


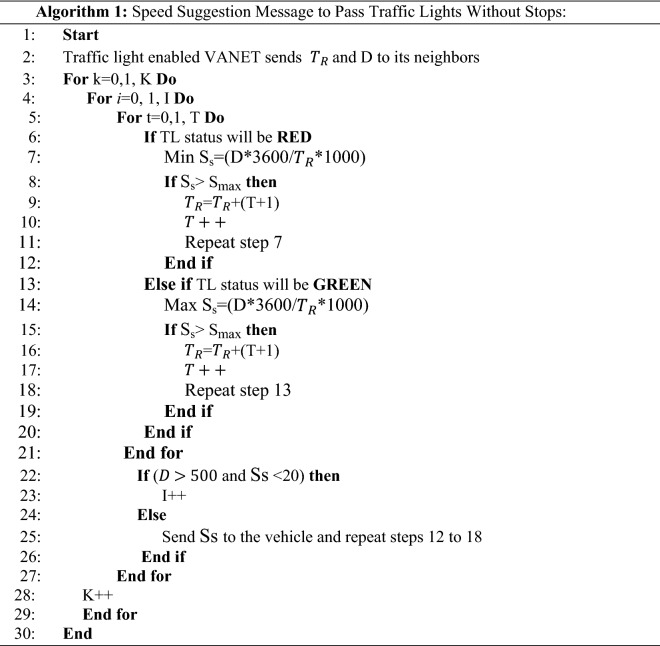


### Dissemination

**Figure 4:**
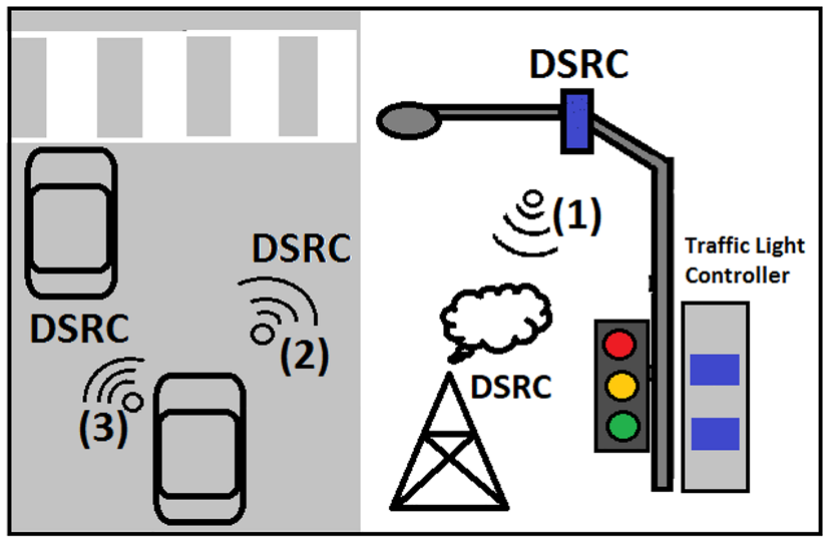
TL, RSU, and vehicle communication using DSRC.

TL recognition methods can be divided into detection and tracking methods. Both are enabled because the TL is sent from the traffic light via VANETs. Figure 4 shows that the traffic light is supported by the VANET. The TL disseminates the traffic light status and timer until it changes to the neighboring RSU and vehicles. The TL status, warnings, and recommended speeds were sent to the vehicle and neighboring vehicles. TL’s status was green, yellow, or red. The warnings included emergencies, accidents, and closed roads.

After computing, the message is transmitted as shown in Algorithm 2. The recommended speed is computed in Section B. Intelligent traffic-light warning messages are defined in Algorithm 2. For traffic light recognition, VANET is a model in which cars move on paved roads. Messages about traffic lights are sent via VANETs to cars. They pass near stable RSUs which are used to define location of vehicles.

Table 3 lists the notation used in Algorithm 2. If there is an emergency, an ambulance will receive a warning for a road branch with a red light. A warning is delivered to automobiles behind them if there is an accident or a closed road route. If a road branch has a red light, a typical car is notified before the red light. If a road branch has a green light, typical vehicles are sent before the green light. If a road branch has a yellow light, typical vehicles are sent before the yellow signal.

**Table 3:** Notations of Algorithm 2 and their descriptions.

Notation	Description
$\mathrm{O}$	Source vehicle region
$\mathrm{N}$	Number of destinations in group member table
S	Status of traffic
W	The warning message about the red light
G	The warning message about the green light
Y	The warning message about the yellow light
T	The warning event time
C	The warning message for closed road


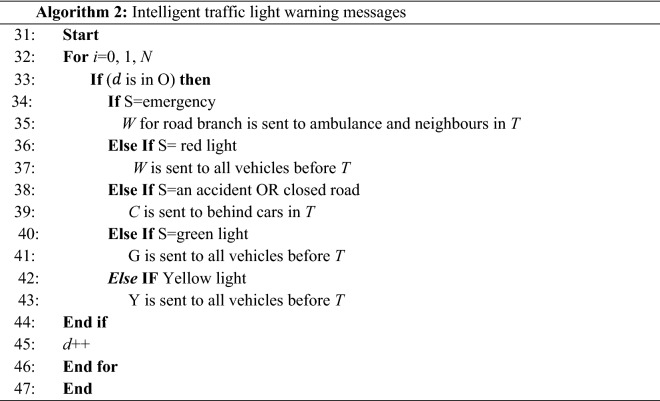


## Performance metrics

In comparison with state-of-the-art approaches, a quantitative evaluation was performed on a common set of traffic lights to investigate the efficiency of the traffic-light status recognition algorithm. Tests were conducted under various lighting and weather conditions. Complex detection methods are used in state-of-the-art detection procedures. The camera was installed on the dashboard and windshield in the front direction in CNN, ICFT, and semi-automatic traffic light detection methods^14,15^. The video set consists of several frames, each of which contains many instances of various traffic signals (red, green, and yellow).

In VANET, the simulation metrics include the packet success rate and end-to-end delay. The packet success rate is defined as the ratio of subsequent packets received by the destination vehicle to the total number of packets sent by the source vehicle. The PDR was calculated using Eq. (4)^21^:

4 $$Packet\,\, success\,\, rate=\frac{R}{S}.$$

R is the number of packets successfully received by the destination vehicle and S is the total number of packets sent by the source vehicle.

The delivery delay is the amount of time it takes for a packet to travel from a source vehicle to a destination vehicle via a vehicular network. Routing process delays such as routing discovery, routing transmission, and retransmission cause this delay, which is calculated by subtracting the source transmission time from the destination arrival time. The average delay for all packets was calculated using Eq. (5)^21^:

5 $$\mathrm{Average\,end}-\mathrm{to}-\mathrm{end\,delay}=\frac{{\sum }_{p=1}^{n}\left({ T}_{R}-{T}_{S}\right)}{n}.$$

## Results and evaluation

The proposed traffic light recognition is disseminated via VANETs. Vehicular network simulation (Veins) is a free and open-source framework for simulating vehicle networks. Veins is used for the proposed VTLR. It is built on top of OMNET +  + and simulates urban mobility (SUMO. SUMO generates VANET data set automatically in the simulation time. The LISA dataset is used for testing camera detection works such as semi-automatic annotation, image processing with CNN, and ICFT.

Vehicular network simulation (Veins) is built on top of OMNET + + and simulates urban mobility (SUMO)^22^. In general, the simulator created an OMNET + + node for each vehicle in the simulation and then matched node movements to vehicle movements in the road traffic simulator (i.e. SUMO). The parameters used in the simulations and their corresponding values are presented in Table 4. The proposed method was tested based on the simulation results of Veins^23^.

**Table 4:** Simulation parameters and values.

Simulation parameter	Value
Vehicles on the roads	50, 100, 150, and 200
Vehicle speed in meters per second (m/s)	10
Range of wireless transmission (m)	200
Dimensions of the playground (mm)	2499 × 2499
Time limits for simulations (s)	5590
Number of beacons (s)	45
Number of traffic lights	35
Number of crossings	15

There are three types of road intersections. The TL is located at an intersection with two or more roads. If only one road at an intersection has two directions, three TLs were constructed. Four TLs are constructed if two roads at an intersection had two directions. There were no traffic lights on one-way streets. On one-way roads, there is no need for traffic lights.

**Figure 5:**
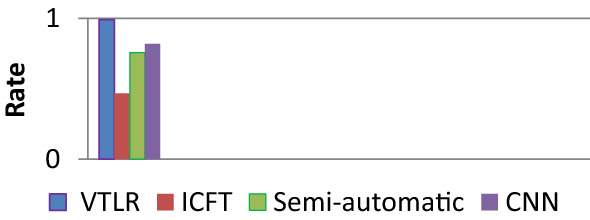
A recognition success rate comparison between VTLR, ICFT, Semi-Automatic, and CNN traffic light detection.

The proposed method is tested under various lighting conditions. It is observed that the proposed method was able to work consistently under different illumination and weather conditions. Figure 5 shows the detection accuracy using delay and the success rate for the CNN, ICFT, and semi-automatic traffic light detection. Table 5 shows implementation results for the success rate and delay.

**Table 5:** Implementation results for the delay and success rate.

Delay and success rate	VTLR	ICFT	CNN	Semi-automatic
Success rate	0.99	0.467	0.8193	0.756
Delay	0.0012	0.075	0.0625	0.125

The success rate on traffic light recognition using VANET is 0.99, respectively. These values were larger than 0.82. In a traffic experiment, the success ratio of ICFT, Semi-Automatic detection**,** and CNN was less than 0.82. The structure of the junction blames for the lack of traffic success in the experiment. The camera can only see selected parts of traffic lights at intersections. Consequently, the traffic signals could not be identified.

**Figure 6:**
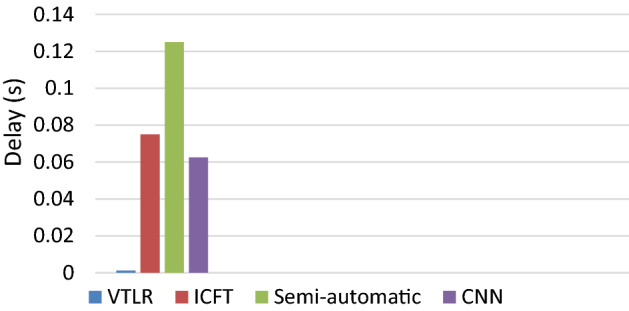
Average delay.

The delay in our protocol is less than 0.002 (s) and outperforms ICFT, semi-automatic detection, and CNN. This is because it exploits stable VANET messages. This result is shown in Fig. 6. The cameras and Neural networks are precise but a lot of time for computations is taken.

**Figure 7:**
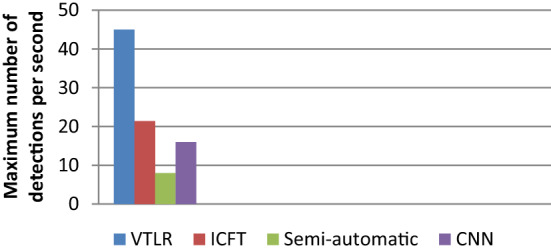
The average number of detections per Second.

Figure 7 shows the localization accuracy using the average number of detections per second for VTLR, ICFT, semi-automatic, and CNN traffic light detection. The traffic light recognition speed using VANET reached 45 detections per second. This speed is larger than 21.4 in ICFT, 8 in semi-automatic detection, and 16 in CNN. This is based on the camera frame rate per second. Table 6 presents the implementation results of the average number of detections per second.

**Table 6:** Implementation results for the maximum number of detections per second.

Maximum number of detections per second	VTLR	ICFT	CNN	Semi-automatic
Number	45	21.4	16	8

## Conclusion

At traffic light junctions, the rapid acceleration, deceleration, and hesitancy of drivers can lead to traffic congestion, accidents, and pollution. Car congestion rises during rush hour in cities. Increasing the road speed and establishing rounds may alleviate traffic congestion and increase the number of accidents. The use of traffic light recognition reduces the number of collisions caused by traffic light systems. TLR are difficult to solve owing to their importance and complexity. A global navigation satellite system that increased car prices. For traffic light detection, semi-automatic annotation was utilized, although tracking was lacking. The data were not collected under adverse conditions. VTLR was proposed to treat these problems and perform semi-automatic annotation detection, ICFT, and image processing with CNN detection in terms of delay, success ratio, and the number of detections per second, according to tests.

## Data Availability

The LISA dataset is used for testing camera detection works such as semi-automatic annotation, image processing with CNN, and ICFT. A huge number of traffic light pictures is taken by front camera of car and stored for computations and is used traffic light detection. The datasets generated and analyzed during the current study are available in the [LISA] repository, [PRESENT WEB LINK TO LISA TRAFFIC LIGHT DATASET]. LISA dataset is widely used in traffic light detection research because it contains 43,007 frames and 113,888 annotated traffic lights in continuous testing and training video sequences. A stereo camera installed on the roof of a vehicle captures sequences while traveling at night and during the day under various lighting and weather conditions.

## References

[1] Laécio R, Firmino N, Glauber G, Soares A, Silva F (2020). Performance evaluation of smart cooperative traffic lights in VANETs. Int. J. Comput. Sci. Eng..

[2] Chen X (2020). Adaptive hybrid model-enabled sensing system (HMSS) for mobile fine-grained air pollution estimation. IEEE Trans. Mob. Comput..

[3] Elsagheer, S. & AlShalfan, K. Intelligent traffic management system based on the internet of vehicles (IoV). *J. Adv. Transport.* **2021**, 10.1155/2021/4037533 (2021).

[4] Sheth K, Patel K, Shah H, Tanwar S (2020). A taxonomy of AI techniques for 6G communication networks. Comput. Commun..

[5] Ziyue L, Zeng Q, Liu Y, Liu J, Li L (2021). An improved traffic lights recognition algorithm for autonomous driving in complex scenarios. Int. J. Distrib. Sens. Netw..

[6] Khatri S, Vachhani H, Shah S, Bhatia J, Chaturvedi M, Tanwar S, Kumar N (2021). Machine learning models and techniques for VANET based traffic management: Implementation issues and challenges. Peer-to-Peer Netw. Appl..

[7] Shirabur, S., Hunagund, S. and Murgd, S. VANET Based embedded traffic control system. In *2020 International Conference on Recent Trends on Electronics* (2020).

[8] Malik S, Sahu P (2019). A comparative study on routing protocols for VANETs. Heliyon.

[9] Ferng H, Tseng Y (2021). An improved traffic rerouting strategy using real-time traffic information and decisive weights. IEEE Trans. Veh. Technol..

[10] Vitas D, Tomic M, Burul M (2020). Traffic light detection in autonomous driving systems. IEEE Consum. Electron. Soc..

[11] Symeonidis G, Groumpos PP, Dermatas E (2019). Traffic Light Detection and Recognition Using Image Processing and Convolution Neural Networks.

[12] Shahista, S. and Khan, A. Detection of the traffic light in challenging environmental conditions. *International Conference on Artificial Intelligence and Soft Computing, EasyChair,* 2021, 5 (2021).

[13] Chen C, Liu B, Wan S, Qiao P, Pei Q (2021). An edge traffic flow detection scheme based on deep learning in an intelligent transportation system. IEEE Trans. Intell. Transp. Syst..

[14] Lee W, Jung K, Kang C, Chang H (2021). Semi-automatic framework for traffic landmark annotation. IEEE Open J. Intell. Transport. Syst..

[15] Wang K, Tang X, Zhao Z, Zhou S, Zhou Y (2021). Simultaneous detection and tracking using deep learning and integrated channel feature for ambint traffic light recognition. J. Ambient Intell. Humaniz. Comput..

[16] Ouyang Z, Niu J, Liu Y, Guizani M (2020). Deep CNN-based real-time traffic light detector for self-driving vehicles. IEEE Trans. Mob. Comput..

[17] Li, Z., Zeng, Q., Liu, Y., Liu, J. & Li, L. An improved traffic lights recognition algorithm for autonomous driving in complex scenarios. *Int. J. Distrib. Sens. Netw.* **17**(5), (2021).

[18] Wang, Q. *et al.* Traffic lights detection and recognition method based on the improved YOLOv4 algorithm. *Sensors* **22**(1), 10.3390/s22010200 (2022). 10.3390/s22010200 PMC8749665 35009743

[19] Saleh A, Darwito HA, Anggraeni AS (2022). Vehicle driver warning systems using road marking and traffic light detection. Am. J. Eng. Res. (AJER).

[20] Iftikhar M, Riaz O, Ali T, Momtaz S (2022). Traffic light detection: A cost effective approach. VFAST Trans. Softw. Eng..

[21] Chanal, P. and Kakkasageri, S. Performance analysis of ant colony based routing approach for VANETs using VanetMobiSim and NS2. In *2019 11th International Conference on Advanced Computing (ICoAC)*, (2019).

[22] Alhaidari, F. & Alerhan, A. A simulation work for generating a novel dataset to detect distributed denial of service attacks on Vehicular Ad hoc NETwork systems. *Int. J. Distrib. Sens. Netw.* **17**(3), (2021).

[23] Weber, J., Neves, M. & Ferreto, T. VANET simulators: An updated review. *J. Braz. Comput. Soc.* **27**(8), 10.1186/s13173-021-00113-x (2021).

